# Quality evaluation of *Cistanche deserticola* and rice wine-steamed products: drying kinetics, intelligent sensory, and chemometrics analysis

**DOI:** 10.3389/fnut.2025.1732810

**Published:** 2026-01-27

**Authors:** Zhangli Jiang, Shiyuan Tang, Xu Wu, Hui Zhang, Xinyi Zhang, Zihan Ma, Xiaohui Bian, Hui Wang, Xin Chai, Yuefei Wang, Zhiying Dou

**Affiliations:** 1 School of Chinese Materia Medica, Tianjin University of Traditional Chinese Medicine, Tianjin, China; 2 Traditional Chinese Medicine Processing Techniques Heritage Base (Tianjin), National Administration of Traditional Chinese Medicine, Tianjin, China; 3 National Inheritance Studio of Expert Chinese Materia Medica, Tianjin, China; 4 State Key Laboratory of Chinese Medicine Modernization, Tianjin University of Traditional Chinese Medicine, Tianjin, China; 5 Haihe Laboratory of Modern Chinese Medicine, Tianjin, China

**Keywords:** chemometrics, *Cistanche deserticola*, color difference meter, drying kinetics, E-nose, E-tongue, processing methods, quality analysis

## Abstract

**Background:**

*Cistanche deserticola* (CD), a functional plant with homology of medicine and food, is used for reinforcing kidney to strengthen yang and loosening bowel to relieve constipation. It is ordinarily processed with rice wine-steamed, which is known as wine-steamed CD (W-CD) to enhance effects in clinical practice. Nevertheless, timely processing of CD is an effective means to ensure quality; the processing techniques also played a crucial role in influencing the quality of CD and its products, which require further investigation. This study aimed to explore suitable drying methods for the efficient production of CDs and W-CDs.

**Methods:**

Herein, the fresh CD is collected and both CD and W-CD are prepared, which all drying mainly included forced-air drying (FAD, at 40, 60, and 80 °C), far-infrared air drying (FID, at 40, 60, and 80 °C), vacuum microwave drying (VMD, at 50, 55, and 60 °C), vacuum freeze drying (VDF), sun-drying (SD) respectively. Furthermore, drying kinetics were employed to analyze drying characteristics, establishing Weibull function models for different processing methods of CD and W-CD. Combining intelligent sensory technologies (E-nose, E-tongue, color difference meter) with texture analyzers, and employing scanning electron microscopy, the trait characteristics and microstructural features were investigated to examine the effects of different drying methods on CD and W-CD. The components content of Echinacoside, Cistanoside A, Tubuloside A, Verbascoside, Isoverbascoside, 2’-Acetylverbascoside, and total polysaccharides are analyzed by high-performance liquid chromatography (HPLC) and ultraviolet–visible spectrophotometry (UV), and the total extracts are also measured. Above those are combined with chemometrics to obtain important factors analysis to differentiate samples of quality.

**Results:**

The Weibull model of drying dynamics is established successfully for CD and W-CD drying processing. The microstructure, rehydration rate (RR, %), and porosity (%) of CD are significantly influenced by rice wine-steamed processing, as are the sweetness (ANS) and content of phenylethyl glycoside, which are also increased. The best drying condition for CD is FAD60-80 °C, and W-CD is FID 40 °C

**Conclusion:**

Our study, which is comprehensive in comparing the quality of CD and W-CD across different drying processes based on “color—odor—taste—component content,” revealed that improving quality can enhance the production of fresh CD. Besides, intelligent sensory technology can provide a foundation for future quality control of CD and W-CD.

## 1 Introduction

**Figure 1:**
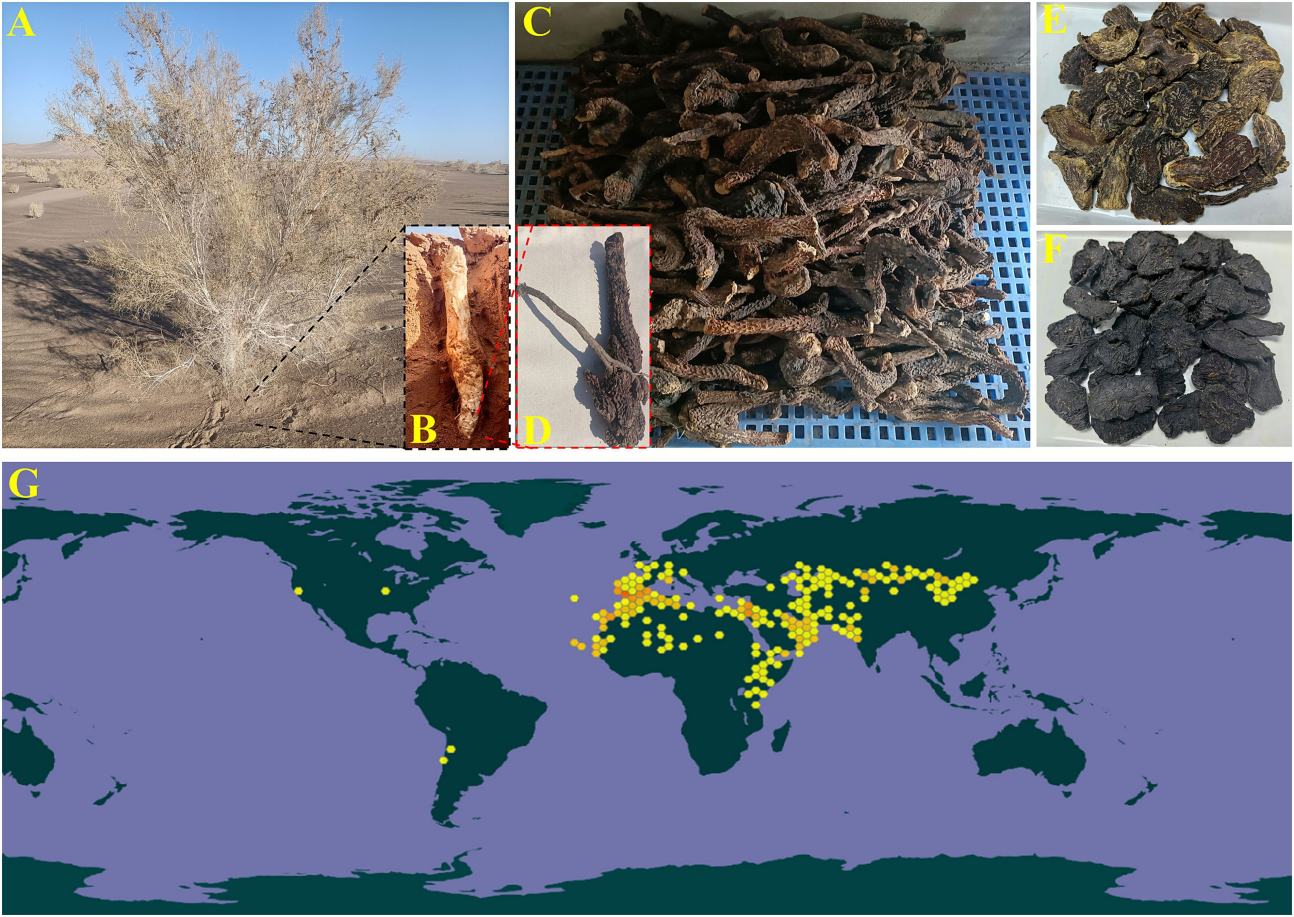
*Haloxylon ammodendron* **(A)**, the plant of *Cistanche deserticola* **(B)**, dry products of *Cistanche deserticola* **(C,E)**, parasitism **(D)**, rice wine-steamed *Cistanche deserticola* **(F)**, and global distribution **(G)**, (www.gbif.org).

*Cistanche deserticola* (*C. deserticola*, CD, Figure 1B), a plant that grows in arid or semi-arid areas, is the dried and scaly fleshy stem of *Orobanchaceae* and is parasitically grown on the hairy root (Figure 1D) of *Haloxylon ammodendron* (Figure 1A). It is also known as *Ròu Cōng Róng* in traditional Chinese medicine (TCM) for tonifying the kidney and yang, benefiting essence and nourishing blood, and moistening the intestine and relaxing bowels (1–3). In 2023, CD was formally included in the *Yaoshi Tongyuan* (medicine and food homology) directory by the National Health Commission of the People’s Republic of China. The medicinal part of CD is the stem, and its two products are listed in the Chinese Pharmacopoeia (ChP), including *Ròu Cōng Róng* (dried CD, Figures 1C,E), and *Jĭu Ròu Cōng Róng* (rice wine-steamed CD or W-CD, Figure 1F). The wild resources of CD are on the verge of extinction; it has been deemed a national second-class protected plant in China (4). Additionally, it’s mainly distributed in the warm and arid areas of the northern hemisphere, from the Iberian Peninsula in Europe, through northern Africa, the Arabian Peninsula in Asia, Iran, Afghanistan, Pakistan, northern India, Kazakhstan, to Inner Mongolia in northwestern China (Figure 1G).

An increasing number of bioactive compounds such as phenylethyl glycosides, iridoid glycosides, lignans, alkaloids, and polysaccharides of CD have been identified in modern research, and are utilized on a large scale, causing its enhanced immunity, slowed aging, alleviated constipation, and anti-inflammatory effects (5–8). Currently, the drying process is one of the indispensable key links in the processing of TCM, which directly affects the quality (9). Modern research shows that different drying methods and processes directly affect the properties, texture, medicinal ingredients, and storage resistance of TCM. Fresh CD has high water content, and its rich carbohydrates and glycosides make it prone to mildew, decay, and enzymatic reactions after harvest, resulting in the loss of core medicinal components (10, 11). Therefore, it is particularly important to compare the quality of CD products under different drying methods and select the optimal processing method and the timely processing of fresh CD. The optimal W-CD drying technology is still unclear. In this study, the applicability and selection principles of different drying methods for CD and W-CD were analyzed from the drying process. At present, the sun drying (SD) and forced air drying (FAD) are commonly used in CD products. As well as far-infrared drying (FID), vacuum microwave drying (VMD), and vacuum freeze drying (VFD), which also have unique drying rates, have also attracted attention in CD processing (12). The SD, FAD of classic drying method and VMD, VFD of new and developing method are employed to investigate drying characteristics and drying kinetics analysis of CD and W-CD. Moreover, the E-nose, E-tongue, and color difference meter of intelligent sensory technology are used in the quality monitoring of foods and TCM to avoid errors caused by subjectivity and to provide scientific characterization of objects (13–16), thereby achieving the purpose of intelligent control based on appearance features. Currently, principal component analysis (PCA), partial least squares discriminant analysis (PLS-DA) and canonical correlation analysis (CCA), and other methods of chemometrics are powerful tools to evaluate complex herbal or some foods products that accomplished then for extracting useful information (17, 18). Above all, intelligent sensors are applied to the quality analysis of CD and W-CD. Combining chemometrics analysis to explore higher efficiency, lower energy consumption, and a more efficient dry method for processing CD and W-CD, which provided high-quality raw materials for CD industrial products.

## 2 Methods and materials

### 2.1 Materials

Rice wine (*Huang jiu*) was purchased by Zhejiang Gu Yue Long Shan Shaoxing Wine Co., Ltd., China. The fresh CD was collected from Alxa League, Inner Mongolia, China, which was identified by Prof. T. X. Li based on each herb documented in China Pharmacopoeia (Part I, 2020 Version).

### 2.2 Chemicals

Echinacoside (Lot#: Y29M10H84490, purity ≥ 98.0%), Cistanoside A (Lot#: P14N9F74960, purity ≥ 98.0%), Tubuloside A (Lot#: Y17J10H93295, purity ≥ 98.0%), Verbascoside (Lot#: Y21A9H59554, purity ≥ 98.0%), Isoacteoside (Lot#: Y23D7H27551, purity ≥ 98.0%), 2’-Acetylverbascoside (Lot#: O13GB163872, purity ≥ 98.0%) were purchased by Shanghai Yuanye Biotechnology Co., Ltd., China. The N-ketone C₆–C₁₆ standard (Lot#: A0114835) was supplied by RESTEK, USA. And the Hydrochloric acid 0.1 mol/L (Art. Nr.: AMCL05.0307.0100, pack: 100 mL) was sourced from Alpha MOS.

### 2.3 Preparation of CD and rice wine-steamed CD

Firstly, the fresh CDs were cleaned and cut into 4 ~ 5 mm slices (China Pharmacopoeia, Part I, 2020 Version). Then, using DHG-9245A-electric hot air drying oven (FAD, Shanghai Yuejin Medical Device Co., Ltd., China) and YHG.300-BS-II-far infrared drying oven (FID, Shanghai Baidian Instrument and Equipment Co., Ltd., China) to dry CD at 40 °C, 60 °C and 80 °C, respectively, which were recorded as CD-FAD40°C, CD-FAD60 °C, CD-FAD80°C, CD-FID40°C, CD-FID60 °C, CD-FID80 °C samples. Meanwhile, CD slices were dried in an RWBZ-08S-Vacuum Microwave Drying Oven (VMD, Nanjing Sunrise Drying Equipment Co., Ltd., China) at 50 °C, 55 °C and 60 °C, and were recorded as CD-VMD 50 °C, CD-VMD 55 °C and CD-VMD 60 °C, respectively. And it’s dried by Alpha 1–2 LD plus-vacuum freeze dryer (VFD, Germany, Marin Christ) at −0.02 mbar, −60 ± 2 °C to obtain sample CD-VFD. Furthermore, the CD slices were dried in sunlight, and the obtained sample was CD-SD. According to our previous study, the W-CD was prepared (11); the process parameters above of CD were also used and were recorded samples as W-CD-FAD40°C, W-CD-FAD60 °C, W-CD-FAD80 °C, W-CD-FID40°C, W-CD-FID60 °C, W-CD-FID80 °C, W-CD-VMD50 °C, W-VMD55 °C, W-CD-VMD60 °C, W-CD-VFD, and W-CD-SD, respectively.

### 2.4 Drying kinetics analysis

#### 2.4.1 Drying rate (DR), dry basis moisture content (M_t_), and moisture ratio (MR) analysis

The weights of CD and W-CD in VFD and SD were measured every 2 h, and in FAD, FID, and VFD, every 1 h, until the moisture content of the sample was less than 12%. Furthermore, the weights of the VMD samples were recorded once every 2 min. Subsequently, the drying rate (DR) is calculated according to the following Equation 1, where Δt (min) denotes the time variation and ΔW (g) represents the moisture loss (19).


$$\mathrm{DR}=\frac{W_{0}-W_{t}}{\mathrm{\Delta t}\;}=\frac{\mathrm{\Delta w}\;}{\mathrm{\Delta t}\;}$$ (1)

The dry basis moisture content (M_t_) of CD and W-CD was calculated as Equation 2, in which the *m*_t_ and *m_e_* represent the mass (g) of the samples at time of “*t*” and “the end point,” respectively.


$$M_{t}=\frac{m_{t}-m_{e}}{m_{e}\;}$$ (2)

Moisture ratio (MR%, g/g) was an important parameter to describe the change of *M_t_* during the drying process, as shown in Equation 3, the *M₀*, *M_t_*, and *M_t_* represent the dry basis moisture content of the sample at time of “0,” “*t*” and “the end point,” respectively (20).


$$\mathrm{MR}=\frac{M_{t}-M_{e}}{M_{0}-M_{e}}\approx \frac{M_{t}}{M_{0}}$$ (3)

#### 2.4.2 Drying curve modeling establishment

The MR was computed and imported Origin 2024 to further characterize ln (MR)-t curve, and it’s aimed to obtain the scale parameters “*α*” and the shape parameters “*β*” as shown in Equation 4 (21). Herein, “*α*” was the scale parameter, and the value was the time required for 63% dehydration of the sample.


$$\mathrm{MR}=\exp[-{\left(t/\alpha \right)}^{\beta }]$$ (4)

#### 2.4.3 Effective moisture diffusion coefficient (D_eff_) and drying activation energy (E_a_) analysis

The *D_eff_* (m^2^/s) was obtained via substituting Fick’s second law into Equation 4 and further to calculate by Equations 5, 6. To describe the accuracy of theoretical moisture diffusion coefficient (D_cal_), Equation 7 was used for calculation, which the “*L*” (m) was the average thickness of CD and W-CD. Furthermore, the drying activation energy (*E_a_*) (kJ/mol) was calculated by using Equations 8, 9. The *D₀* (m^2^/s) was the frequency factor of the effective moisture diffusion coefficient, *R* [8.314 J/(mol·K)]was the gas constant, and “*T*” was the temperature in Kelvin (T_K_ = T_°C_ + 273.15).


$$\mathrm{MR}=\frac{8}{\pi^{2}}\exp(-\frac{\pi^{2}D_{\mathrm{eff}}t}{L^{2}})$$ (5)

$$\text{lnMR}=\ln\frac{8}{\pi^{2}}-\frac{\pi^{2}D_{\mathrm{eff}}t}{L^{2}}$$ (6)

$$D_{\mathrm{cal}}=\frac{L^{2}}{\alpha }$$ (7)

$$D_{\mathrm{eff}}=D_{0}\exp(\frac{-\mathrm{Ea}}{RT_{K}})$$ (8)

$$\text{lnMR}=\ln\frac{8}{\pi^{2}}-\frac{\pi^{2}D_{\mathrm{eff}}t}{L^{2}}$$ (9)

### 2.5 Texture and structure characteristic analysis

#### 2.5.1 Microstructure observation

Observation of the CD and W-CD microstructures of different samples was conducted in accordance with our previous study (22), following these specific steps: samples were cut into 1 cm square blocks, first immersed in methanol for 1 hour, then sequentially immersed in 30, 50, 70, and 90% ethanol solutions for 30 min each, and finally immersed in ethanol for a further 30 min (twice). Lastly, immerse the sample in tert-butyl alcohol for 30 min (twice), then vacuum-freeze-dry for 5 h. Above these samples were placed on the holder of the high-resolution field emission scanning electron microscope (ZEISS, Germany, Merlin Compact). Set the voltage to 3.00 kV and adjust the magnification to 1,000 × to capture the image.

#### 2.5.2 Porosity analysis

ImageJ was used to quantitatively analyze the porosity of the CD and W-CD samples from microstructure images.

#### 2.5.3 Rehydration ratio (RR) characteristics

The samples were soaked in water at a ratio of 1:10 (v/v) for 1 h, subsequently dried by blotting the surface with absorbent paper, and weighed. The rehydration ratio (RR, %) was calculated according to Equation 10 (23), in which *W_b_* (g) and *W_a_* (g) represented the mass of different samples before and after rehydration, respectively.


$$\mathrm{RR}=\frac{W_{a}}{W_{b}}$$ (10)

#### 2.5.4 Characteristics of hardness

The EZ-LX-texture analyzer (Shimadzu Corporation, Japan), equipped with a 2 mm-diameter cylindrical probe, was used to analyze the hardness of CD and W-CD. After calibration, the stroke was set to 13 mm, with downward and upward speeds of 1.0 mm/s, and the speed was set to 0.5 mm/s to test the samples.

### 2.6 HPLC, total polysaccharides, and total extracts analysis

According to the Chinese pharmacopoeia (Part IV, 2020 Version), assayed total extracts of CD and W-CD. Furthermore, the HPLC and total polysaccharides analyses were referenced to our previous study, described and slightly modified (11). Briefly, the samples of CD and W-CD powder (0.5 g) were accurately weighed, soaked in a 50% methanol solution (1:100, w/v) for 30 min, and extracted for 40 min by ultrasound (150 W, 40 kHz). These extracts were filtered (0.22 μm). Each reference substance solution with a concentration of 3 mg/mL was prepared for HPLC analysis, including echinacoside, cistanoside A, tubuloside A, verbascoside, Isoacteoside, and 2′-acetylverbascoside. A 1260 high-performance liquid chromatograph (Agilent Technologies, USA) was used to determine the chemical constituents. An Agilent Eclipse XDB-C_18_ chromatographic column (250 × 4.6 mm, 5 μm) was employed. The flow rate was set to 1.0 mL/min, the column temperature was set at 30 °C, and the injection volume was 10 μL. Both the 0.1% formic acid water (A) and acetonitrile (B) were used as the mobile phase, which was as follows: 0–10 min, 9–15% B; 10–40 min, 15–26% B; 40–45 min, 26–9% B; 45–48 min, 9% B. Furthermore, 0.50 g samples powders were added 25 mL alcohols (80%), extracted for 30 min by ultrasound (60 °C, 150 W, 40 kHz), centrifuged (4,000 rpm) to 10 min, filtered and collected filter residue, added 25 mL water again, extracted for 30 min by ultrasound (80 °C, 150 W, 40 kHz), centrifuged (4,000 rpm) to 10 min, extracted to repeat twice and combined, finally for using determination of polysaccharide by phenol-sulfate acid method. Methodological evaluations of HPLC and UV were presented in Supplementary Tables S1–S8.

### 2.7 Intelligent sensory analysis of “color—odor—taste”

#### 2.7.1 Color difference meter analysis

CM-5-Differential Refractometer (Konica Minolta, Japan) was optimized for color (L^*^-value, a^*^-value, b^*^-value) measurement of CD and W-CD, which the L^*^-value represents the brightness value, ranging from black (0) to white (100), a^*^-value represents the red-green value, ranging from green (−60) to red (60), and b^*^-value represents the yellow-blue value, ranging from blue (−60) to yellow (60) (23). Then, the performance color of Differential Refractometer was calibrated, light source was used D65, observed angle of 2°, and illumination aperture was 30 mm. The color difference (ΔE^*^) between the samples was calculated by using Equation 11.


$$\Delta E^{*}=\sqrt{\Delta L^{*2}+\Delta a^{*2}+\Delta b^{*2}}$$ (11)

#### 2.7.2 E-nose analysis

The different samples of CD and W-CD were pulverized through a No.4 sieve (China Pharmacopoeia, Part I, 2020), and 1.0 g of powder was placed in a 20 mL headspace vial for analysis of volatile components (VOCs), which was achieved using ASTREE II-Electronic Nose (Alpha MOS, France). The parameters for the analysis are shown in Table 1. Separation columns, both MXT-5 and MXT-1701, were used. All analyses were conducted 3 times. The Alphasoft V14.2 software was used to identify VOCs in samples. An N-ketone C₆–C₁₆ standard mix was used to calculate the Kovats indices using the Arochembase database.

**Table 1:** Electronic nose detection conditions.

Items	Conditions	Set parameters
Headspace sampling	Sample volume	1.0 g
	Heating and oscillation temperature	60 °C
	Heating and oscillation time	15 min
Injection	Injection volume	4,000 μL
	Injection speed	200 μL/min
	Injection port temperature	200 °C
	Injection port pressure	10 kPa
	Injection duration	25 s
Trapping trap	Initial temperature	40 °C
	Split	20 mL/min
	Trapping duration	30 s
Column oven	Initial temperature	40 °C (3 s)
	Programmed temperature	1°C/s^−1^---80 °C (0) s
		2 °C/s^−1^---204 °C (10) s
		1°C/s^−1^---250 °C (20) s
	Acquisition time	181 s
Detector	Detector temperature	260 °C
	FLD gain	12

#### 2.7.3 E-tongue analysis

The samples of CD and W-CD powder (1.0 g) were soaked in water (25 mL) for 30 min and then ultrasonic (150 W, 40 Hz, 30 °C) extracted for 20 min, centrifugation (3,500 rpm, 15 min), to collect supernatant. The ASTREE II -electronic tongue (Alpha MOS, France) was pre-calibrated with a 0.01 mol/L hydrochloric acid solution and distilled water. Detection time was 120 s, and the test was conducted continuously 5 times. The last three results of sourness (AHS), sweetness (ANS), bitterness (SCS), saltness (CTS), and freshness (NMS) were used for data analysis.

### 2.8 Chemometrics analysis

All the above detection indexes were imported into SIMICA 14.0 for PLS-DA analysis. The permutation test (200 times) was used to assess whether the model was over-fitting, and different factors were identified by Variable Importance Projection (VIP) > 1 and *p* < 0.05. Furthermore, the differences between the samples were compared, and the dried processing methods were comprehensively evaluated.

### 2.9 Correlation analysis and analytic hierarchy process (AHP) analysis

All indicators of CD and W-CD in this study were used in correlation analyses, and the effects of different drying methods (SD, FAD, FID, VMD, and VFD) on the appearance and internal compounds of the product were studied. A *p* < 0.05 was considered to indicate a significant correlation in this study. Additionally, the drying time (*Y_1_*), the content of Echinacoside (*Y_2_*), Verbascoside (*Y_3_*), total extracts (*Y_4_*), total polysaccharides (*Y_5_*), Cistanoside A (*Y_6_*), Tubuloside A (*Y_7_*), Isoacteoside (*Y_8_*), and 2’-Acetylverbascoside (*Y_9_*) were mainly collected for AHP analysis (24), the positive index was calculated by 𝑦 = (𝑥 − 𝑥_min_)/(𝑥_max_ − 𝑥_min_), and negative index was calculated by 𝑦 = (𝑥_max_ − 𝑥)/(𝑥_max_ − 𝑥_min_) to comprehensive analysis to determine the best drying process for CD and W-CD.

### 2.10 Statistical analysis

All statistical analyses were performed using Prism10.1.2. Continuous variables that were normally distributed were presented as mean ± standard deviation (mean ± SD). Repeated measures ANOVA was used for differences, and a *p*-value < 0.05 was considered statistically significant.

## 3 Results

### 3.1 Drying characteristics of CD and rice wine-steamed CD

**Figure 2:**
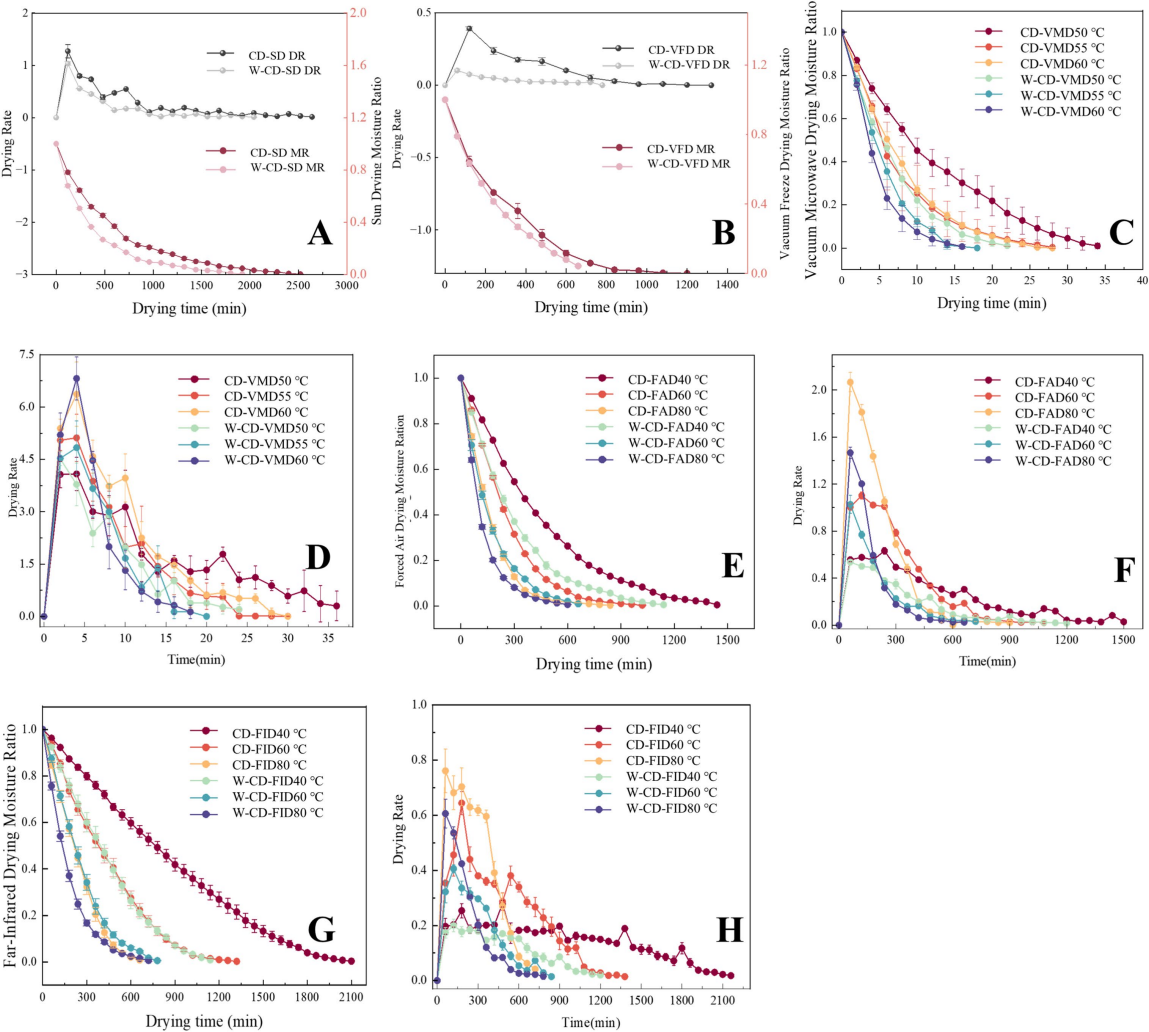
Drying characteristics of CD and rice wine-steamed CD. MR and DR curve over time of SD samples **(A)**, MR and DR curve over time of VFD samples **(B)**, MR and DR curve over time of VMD samples **(C,D)**, MR and DR curve over time of FAD samples **(E,F)**, MR and DR curve over time of FID samples **(G,H)**.

The DR and MR curves of CD and W-CD during the SD process were assessed (Figure 2A), in which the DR increased with temperature rise, and the drying time of W-CD was 22.7% shorter than that of CD. In VFD, the DR and MR curves of CD and W-CD were assessed during the process (Figure 2B), showing that the water loss rate of W-CD was faster than that of CD (40.0%). Compared with the SD samples, the W-CD in the VFD was shortened by 72.7%, and the CD was shortened by 54.5%. Additionally, the MR (Figure 2C) and DR (Figure 2D) curves of CD and W-CD in VMD showed that the water loss rate of W-CD exceeded that of CD at 50 °C, 55 °C, and 60 °C, respectively. And the drying time before and after rice wine-steaming of CD was shortened by 36.8% at 50 °C, 33.3% at 55 °C, and 35.7% at 60 °C. The drying time of VMD was 97.1% ~ 98.6% shorter than that of SD, significantly improving drying efficiency. The higher the drying power and temperature, the shorter the time required to achieve the same drying degree. Similarly, the MR and DR curves in both FAD (Figures 2E,F) and FID (Figures 2G,H) of CD and W-CD during the process were also assessed, with the order of water loss rate as follows: CD-FID80°C > W-CD-FAD80°C > W-CD-FAD60 °C > CD-FAD60 °C > W-CD-FAD40°C > CD-FAD40°C. The drying time of W-CD was 20.0% shorter than that of CD in FAD at 40 °C, 33.3% shorter than CD in FAD at 60 °C, and 26.7% shorter than CD in FAD at 80 °C. At 60 °C, VMD was the fastest, followed by FAD and FID. Based on these results, we found that different drying methods have distinct advantages in the processing of CD and W-CD. Therefore, Weibull model fitting was further performed to study the D_eff_ (m^2^/s) and E_a_ (kJ/mol).

**Figure 3:**
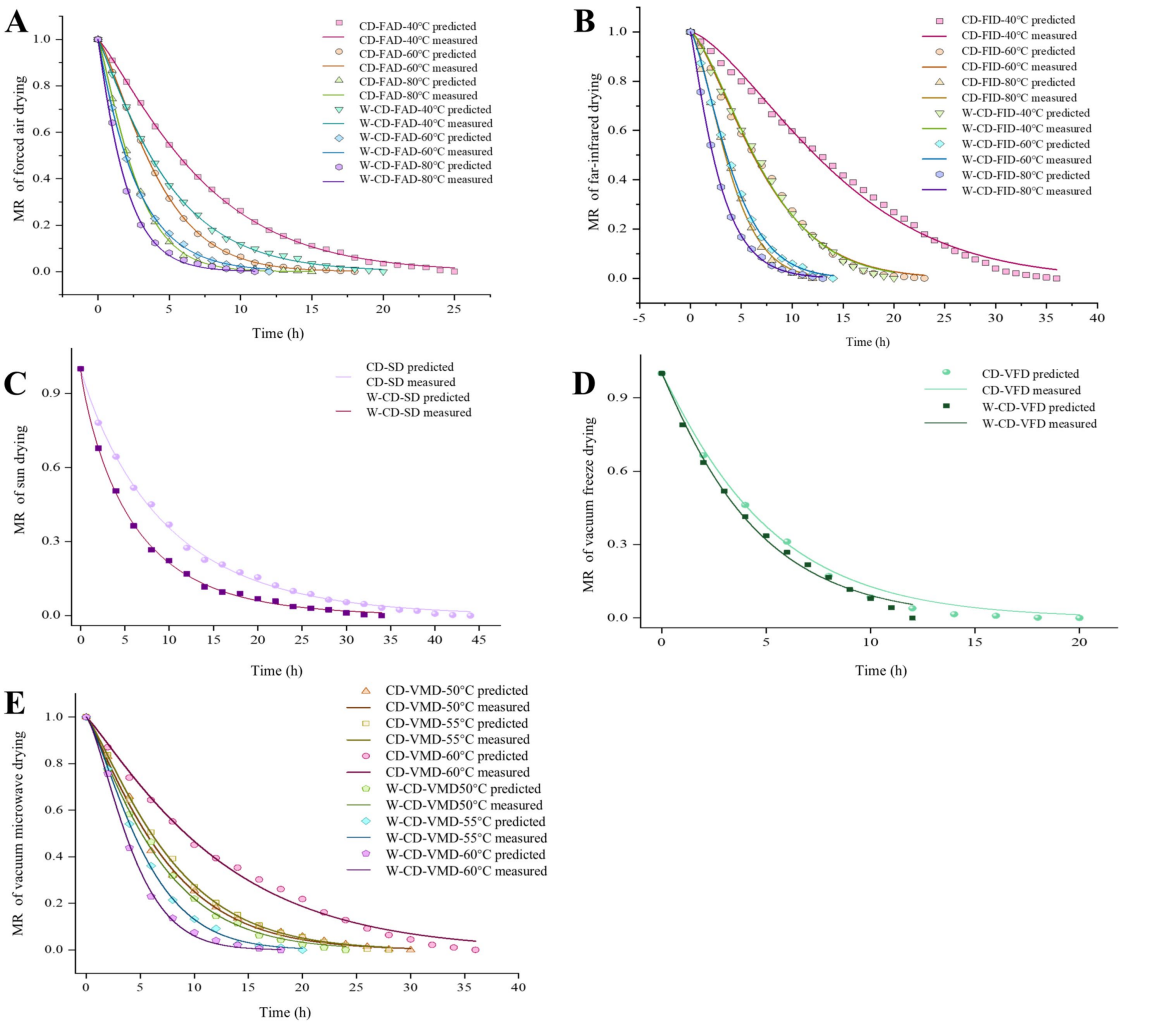
Weibull distribution function model fitting curves of forced air drying **(A)**, far-infrared drying **(B)**, sun drying **(C)**, vacuum freeze drying **(D)**, and vacuum microwave drying **(E)**.

The Weibull functional model was fitted to the drying data (Table 2; Figure 3), and the R^2^ of different drying methods were 0.9926 ~ 0.9998, the root mean square error (RMSE) was 0.0034 ~ 0.0266, and *x*^2^ were 1.73 × 10^−5^ ~ 7.50 × 10^−4^. The higher R^2^ (> 0.99), the smaller the sum of squared deviations (*x*^2^) and RMSE, suggesting that the fitting results of the Weibull function were suitable for the analysis of CD and W-CD drying process, and further to prediction analysis (25). Moreover, the *α* values of the samples ranged from 4.73 to 947.19 min, and the smaller *α* value corresponded to the shorter drying time. The *β* value ranges from 0.86 to 1.47, which determines the degree of curvature in the fitting curve and the convergence behavior of the tail, and affects the overall shape of the drying process. The D_eff_ values of different drying equipment at 60 °C were CD-VMD > W-CD-VMD > W-CD-FID = W-CD-FAD > CD-FAD> CD-FID2. The VMD showed significantly higher D_eff_ than other drying methods due to its unique drying mechanism. The E_a_ was the minimum energy required for moisture to migrate from the interior of the material to the surface and evaporate during drying. It was used to compare CD and W-CD under different drying equipment conditions. For VMD processing, E_a_ changed slightly, indicating that the energy required to evaporate water from the inside before and after wine steaming for CD was almost the same. However, the E_a_ of FAD and FID was nearly halved, as shown in Table 2. Both FAD and FID show lower energy consumption.

**Table 2:** The Weibull model factors, D_eff_, and D_cal_ of drying methods.

Samples	*α* (min)	*β* (min)	*x* ^2^	RMSE	R^2^	D_eff_ (m^2^ /s)	D_cal_ (m^2^ /s)	E_a_ (kJ/mol)
CD-FAD40 °C	459.96	1.2	5.26 × 10^−5^	0.0070	0.9994	8.11 × 10^−11^	5.80 × 10^−10^	16.14
CD-FAD60 °C	269.46	1.31	1.73 × 10^−5^	0.0039	0.9998	1.46 × 10^−10^	9.90 × 10^−10^	
CD-FAD80 °C	168.02	1.22	3.64 × 10^−5^	0.0056	0.9996	1.62 × 10^−10^	1.59 × 10^−9^	
CD-FID40 °C	947.19	1.47	7.50 × 10^−4^	0.0266	0.9926	6.48 × 10^−11^	2.82 × 10^−10^	21.13
CD-FID60 °C	479.43	1.4	4.46 × 10^−4^	0.0202	0.9958	1.13 × 10^−10^	5.56 × 10^−10^	
CD-FID80 °C	263.39	1.47	4.59 × 10^−4^	0.0205	0.9962	1.62 × 10^−10^	1.01 × 10^−9^	
CD-VMD50 °C	12.7	1.14	6.07 × 10^−4^	0.0233	0.9935	3.24 × 10^−9^	2.10 × 10^−8^	39.36
CD-VMD55 °C	7.23	1.31	5.07 × 10^−4^	0.0211	0.9950	4.70 × 10^−9^	3.52 × 10^−8^	
CD-VMD60 °C	8.16	1.21	6.43 × 10^−5^	0.0075	0.9994	5.03 × 10^−9^	3.27 × 10^−8^	
CD-VFD	290.59	1.15	3.88 × 10^−4^	0.0171	0.9964	1.46 × 10^−10^	8.73 × 10^−10^	-
CD-SD	578.58	0.93	1.39 × 10^−4^	0.0112	0.9982	4.86 × 10^−11^	4.61 × 10^−10^	-
W-CD-FAD40 °C	304.74	1.12	4.55 × 10^−5^	0.0064	0.9995	1.13 × 10^−10^	8.75 × 10^−10^	8.35
W-CD-FAD60 °C	165.15	1.03	3.38 × 10^−5^	0.0053	0.9997	1.62 × 10^−10^	1.61 × 10^−9^	
W-CD-FAD80 °C	119.83	1.07	1.52 × 10^−4^	0.0113	0.9984	1.62 × 10^−10^	2.23 × 10^−9^	
W-CD-FID40 °C	483.27	1.44	4.18 × 10^−4^	0.0194	0.9961	9.73 × 10^−11^	5.52 × 10^−10^	11.96
W-CD-FID60 °C	278.82	1.37	9.31 × 10^−5^	0.0090	0.9992	1.62 × 10^−10^	9.56 × 10^−10^	
W-CD-FID80 °C	182.56	1.13	2.32 × 10^−5^	0.0045	0.9998	1.62 × 10^−10^	1.46 × 10^−9^	
W-CD-VMD50 °C	7.01	1.17	2.02 × 10^−4^	0.0131	0.9981	5.35 × 10^−9^	3.80 × 10^−8^	39.02
W-CD-VMD55 °C	5.83	1.34	1.30 × 10^−4^	0.0103	0.9989	8.27 × 10^−9^	4.58 × 10^−8^	
W-CD-VMD60 °C	4.73	1.38	1.95 × 10^−4^	0.0132	0.9984	8.27 × 10^−9^	5.64 × 10^−8^	
W-VFD	265.19	1.06	5.60 × 10^−4^	0.0218	0.9941	1.13 × 10^−10^	1.01 × 10^−9^	-
W-SD	362.32	0.86	7.41 × 10^−5^	0.0081	0.9990	6.48 × 10^−11^	7.36 × 10^−10^	-

“-” means not involved.

### 3.2 Microstructure observation of CD and rice wine-steamed CD

**Figure 4:**
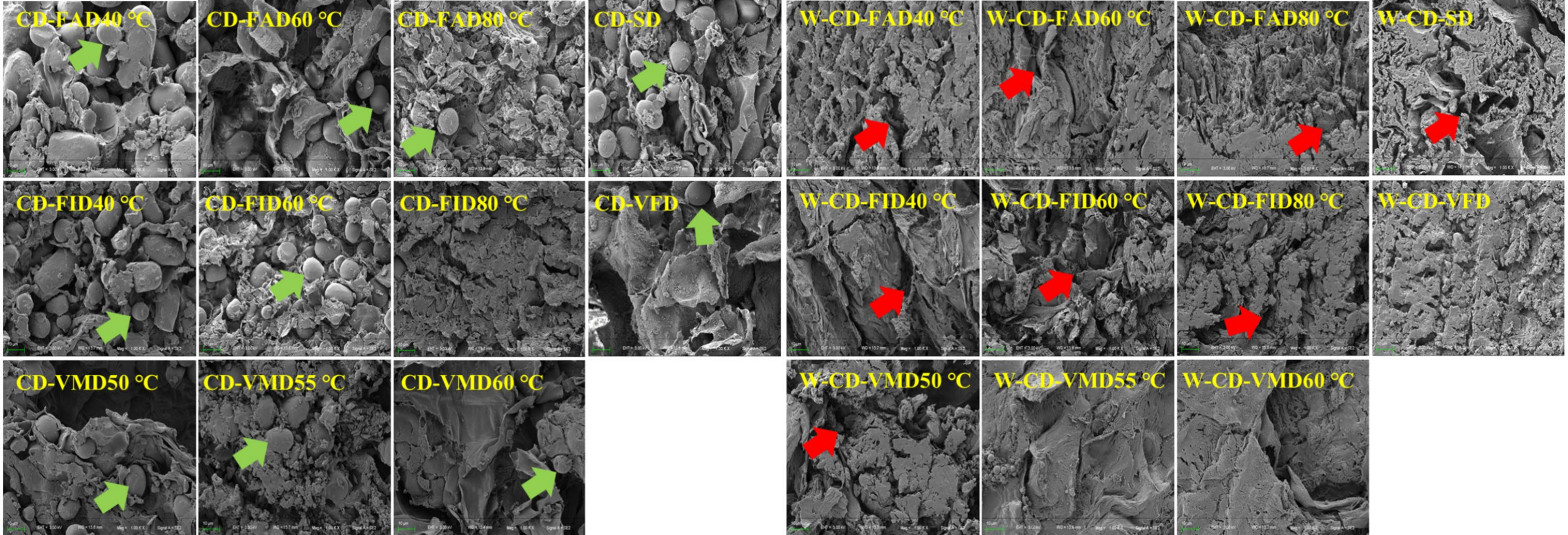
Microstructure images of CD and rice wine-steamed CD (1000×). The green arrow refers to the starch granules, and the red arrow refers to the cavity structure.

Samples of CD and W-CD under different drying processes were used to observe the microstructure and analyze their differences in texture (Figure 4). It can be observed that in CD, the starch granules are gradually covered by the gel layer, and the surface structure becomes more compact as the temperature increases. This may be due to the high temperature, which causes the water in the CD cells to diffuse to the surface, where it interacts with the surface amylopectin granules, resulting in gelatinization (26). VFD technology can preserve the original structure of the sample to the greatest extent possible; the structure of the CD in VFD was in a loose state with clearly visible starch granules. Similarly, the starch granules of CD in FAD, FID, and VMD exhibit this structural trend, while some starch granules have been gelatinized. However, the structure of W-CD shows obvious changes; the starch granules were gelatinized to the point that they were difficult to observe in different dry methods. And the structures were obviously cracked, which may be one reason for the quality difference among them. It was suggested that CD was significantly influenced by the rice wine-steaming process. After that, the RR (%) and porosity (%) were further studied to investigate the effects of different drying processes on CD and W-CD.

### 3.3 Porosity and rehydration rate of CD and rice wine-steamed CD

The results are shown in Table 3, where the RR of CD were VFD > VMD ≈ FID > SD > FAD, and of W-CD were VMD > FAD > VFD ≈ SD ≈ FID. The structure of W-CD has changed evidently. Furthermore, the porosity was calculated and presented in Table 3. Rice wine-steamed proccessing CD can cause significant changes, consistent with the microstructure results. This processing method for W-CD products helps to maintain good water exchange and absorption capacity. Those samples of CD and W-CD from different processing methods were further analyzed by HPLC and UV to investigate the effect of drying methods on the content of components.

**Table 3:** Porosity and rehydration ratio of different samples.

Samples	RR (%)	Porosity (%)	Samples	RR (%)	Porosity (%)
CD-FAD40 °C	42.89	34.21	W-CD-FAD40 °C	47.08	38.43
CD-FAD60 °C	48.48	47.15	W-CD-FAD60 °C	54.65	42.86
CD-FAD80 °C	45.83	45.09	W-CD-FAD80 °C	57.24	45.99
CD-FID40 °C	76.82	39.83	W-CD-FID40 °C	40.03	45.71
CD-FID60 °C	63.79	44.10	W-CD-FID60°C	38.74	46.27
CD-FID80 °C	45.83	34.14	W-CD-FID80 °C	50.22	40.56
CD-VMD50 °C	63.67	45.63	W-CD-VMD50 °C	61.06	45.84
CD-VMD55 °C	70.77	41.67	W-CD-VMD55 °C	70.67	39.98
CD-VMD60 °C	72.31	41.77	W-CD-VMD60°C	70.95	26.37
CD-VFD	103.96	52.22	W-VFD	49.12	31.61
CD-SD	52.30	48.08	W-SD	48.61	42.43

### 3.4 Content determination, total polysaccharides, and extracts of CD and rice wine-steamed CD

**Figure 5:**
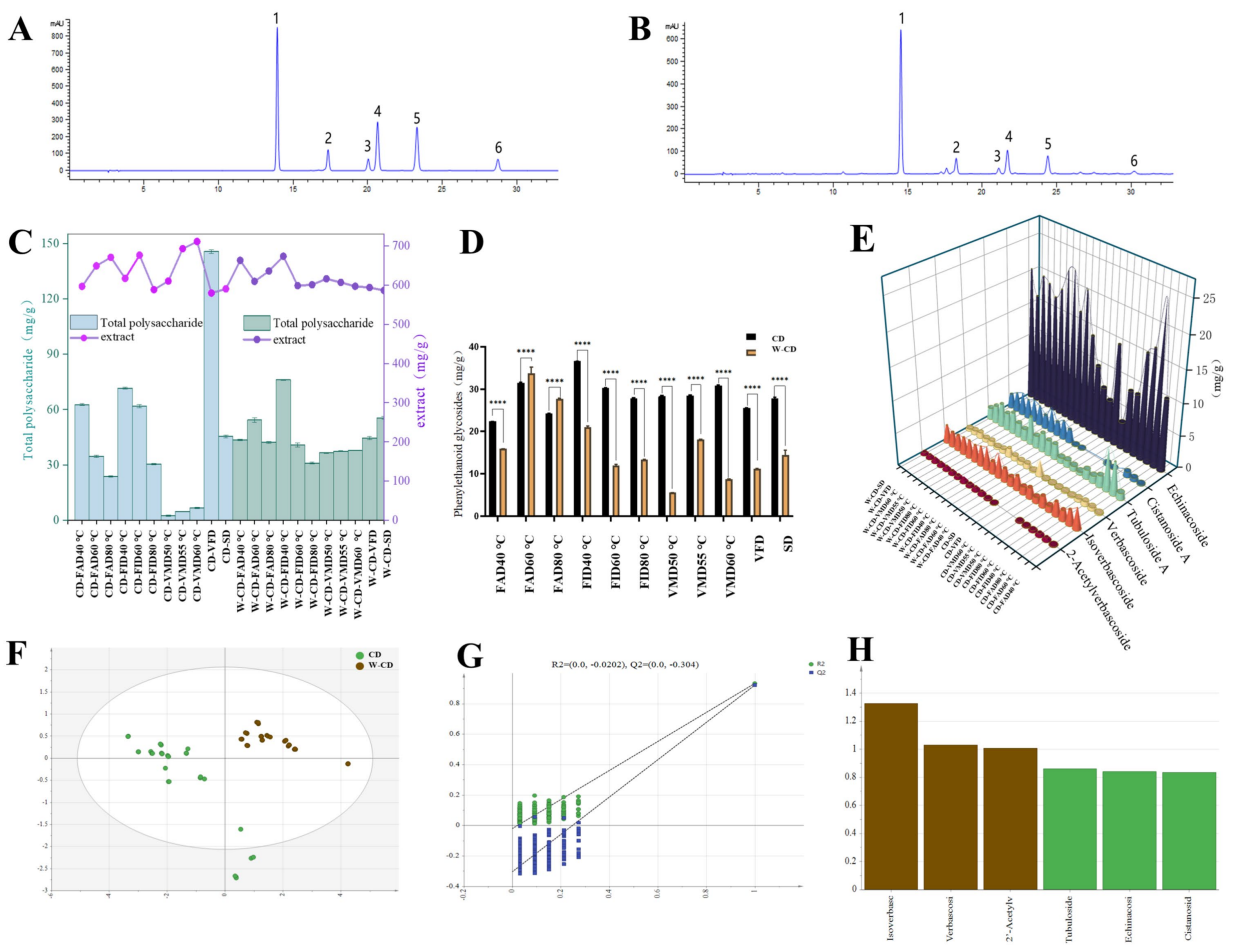
The content analysis of different processing methods of CD and rice wine-steamed CD. The HPLC plots of standard (**A**, 1: Echinacoside; 2: Cistanoside A; 3: Tubuloside A; 4: Verbascoside; 5: Isoacteoside; 6: 2’-Acetylverbascoside) and samples **(B)**; total polysaccharides and total extracts **(C)**; phenylethanoid glycosides **(D,E)**; the PLS-DA analysis **(F)**, permutation test **(G)**, and VIP analysis **(H)** of CD and W-CD (*p < 0.05, **p < 0.01, ***p < 0.001, ****p < 0.0001).

To further investigate how the dry method to effect CD and W-CD, the content of echinacoside, cistanoside A, tubuloside A, verbascoside, Isoacteoside, 2′-acetylverbascoside, total polysaccharide and total extracts of CD and W-CD were performed (Figure 5). By comparing the standard solution (Figure 5A), the target compounds in the solution to be tested were well separated (Figure 5B). The phenylethyl glycoside content was calculated and shown in Figures 5D,E, which the phenylethyl glycoside content increased significantly after the process of rice wine-steamed of CD. In VMD processing and compared with CD, the content of W-CD increased by 80.33% (40 °C), 36.58% (60 °C), and 71.67% (80 °C), respectively. However, excessive microwave power or long-term microwave exposure can produce thermal hot spots, resulting in the degradation of thermosensitive compounds (27). Additionally, during CD drying, the phenylethyl glycoside content of FAD was higher than that of SD, especially for FAD80 °C. In drying W-CD, the phenylethyl glycoside content of FID and VMD was also higher than that of SD. As for the phenylethyl glycoside conversion trend, the conversion of components was promoted after rice wine-steaming, and different drying conditions had little effect on it. Compared with CD content, the fluctuation range was smaller and more stable. The results for total polysaccharide and total extract contents of CD and W-CD are shown in Figure 5C. Polysaccharide content was better retained in the VFD-processed CD, whereas it decreased in dried by FAD and FID. Currently, the total extracts of CD in VMD were higher than FAD and FID, and the total extracts of W-CD were highest in FID at 40 °C.

### 3.5 Appearance properties of CD and rice wine-steamed CD

#### 3.5.1 E-nose of odor analysis

**Figure 6:**
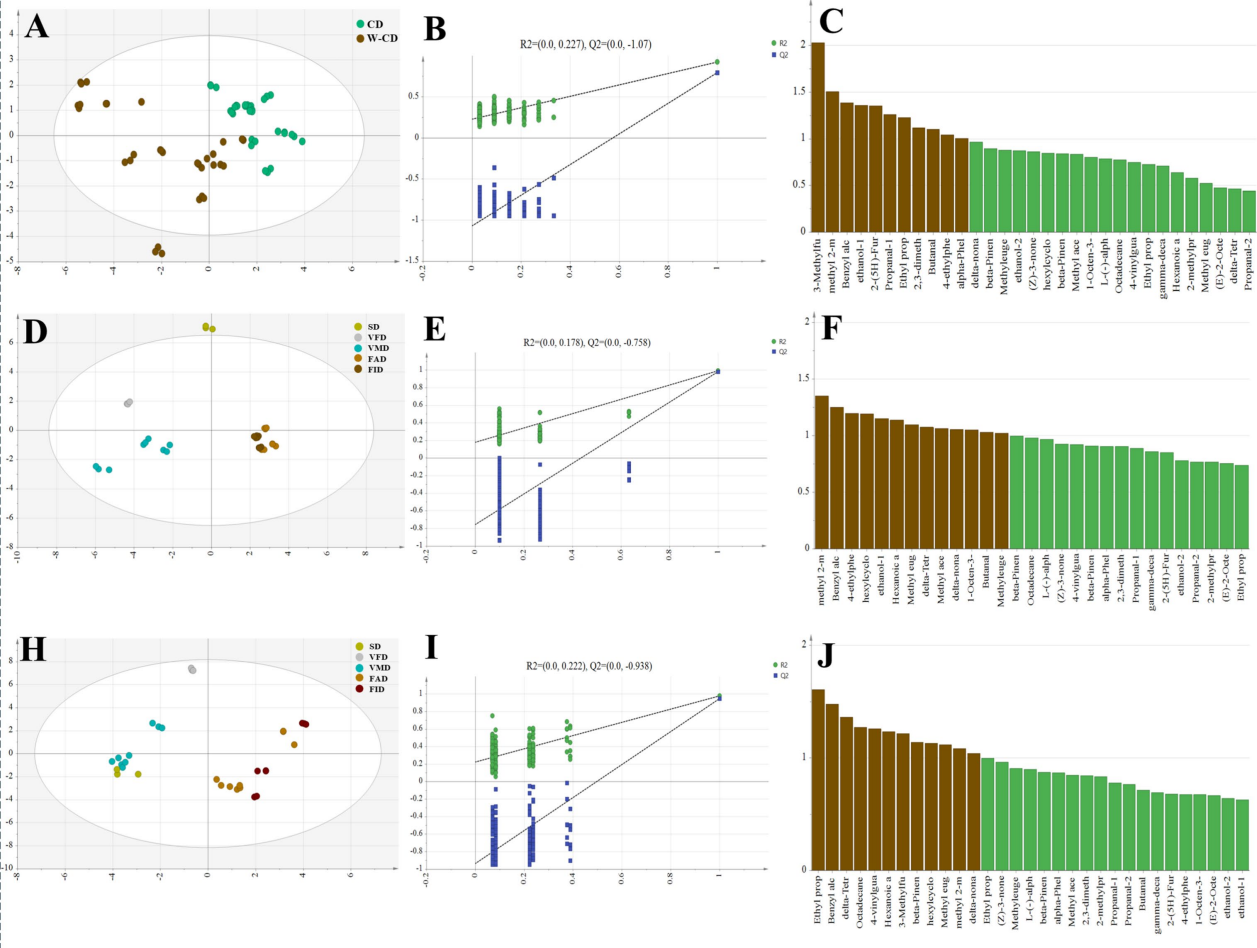
VOC components of different processing methods of CD and rice wine-steamed CD. The PLS-DA analysis **(A)**, permutation test **(B)**, and VIP analysis **(C)** of CD and W-CD; PLS-DA analysis **(D)**, permutation test **(E)**, and VIP analysis **(F)** of CD in different dry methods; PLS-DA analysis **(H)**, permutation test **(I)**, and VIP analysis **(J)** of W-CD in different dry methods.

To explore the effect of different drying processes on the VOCs of CD and W-CD, the E-nose was used to detect them. A total of 49 VOCs were identified, as shown in Table 4, of which two compounds have no obvious odor. The aroma profile was evaluated using the following descriptors: floral, fruity, grassy, minty, camphor, woody, sweet, and spicy (28), such as (*E*)-Cinnamaldehyde described as sweet spice, candy cinnamon red hots warm, 1, 8-cineole was eucalyptus herbal camphor medicinal, and 1-Hexadecanol was characteristic for waxy, clean, greasy floral, and oily. Moreover, those VOCs data were imported into SIMCA 14.1 to clarify the impact of different treatment methods on odors of CD and W-CD (Figure 6). First, rice wine-steamed before and after of CD were evaluated via OPLS-DA (R^2^_x_: 0.798, R^2^_y_: 0.923, Q^2^: = 0.794) that was suggested wine-steamed changed significantly (Figure 5A). The replacement test results (R^2^: 0.227, Q^2^: −1.07) show that the model fits well without over-fitting (Figure 6B). The VIP score reflects the weight of the differences between odor groups and methyl 2-methylbutanoate, benzyl alcohol, ethanol, 2-(5H)-Furanone, propanal, ethyl propanoate of VIP > 1 were considered differential markers (Figure 6C). Then, the PLS-DA model of CD in different drying methods were evaluated (R^2^_x_: 0.939, R^2^_y_: 0.976, Q^2^: 0.938) that the CD’s odor of different drying methods also has changed (Figure 6D), the replacement test results (R^2^: 0.178, Q^2^: 0.758) show that the model fits well without over-fitting (Figure 6E), methyl 2-methylbutanoate, benzyl alcohol, 4-ethyl phenol, ethanol, hexanoic acid were differential markers (Figure 6F), and CD of FAD and FID have similar odorous substances. As shown by PLS-DA analysis, the odor of W-CD for different drying methods has changed (Figure 6H). These W-CD can be roughly divided into two categories: one part was VFD, VMD, SD, and the other was FAD, FID. The replacement test results (R^2^: 0.222, Q^2^: −0.938) indicate that the model fits well without over-fitting (Figure 6I). Ethyl propanoate, benzyl alcohol, delta-tetradecalactone, octadecane, and 4-vinylguaiacol were differential markers (Figure 6J).

**Table 4:** Information on VOCs for E-nose.

No.	Compounds	Formula	CAS#	Descriptions
1.	(*E*)-Cinnamaldehyde	C_9_H_8_O	14371-10-9	Sweet spice candy cinnamon red hots warm
2.	1, 8-cineole	C_10_H_18_O	470-82-6	Eucalyptus herbal camphor medicinal
3.	1-Hexadecanol	C_16_H_34_O	36653-82-4	Waxy clean greasy floral oily
4.	D-(+)-alpha-pinene	C_10_H_16_	7785-70-8	Harsh terpene aromatic minty
5.	L-(−)-alpha-pinene	C_10_H_16_	7785-26-4	Sharp, warm, resinous, fresh pine
6.	2-(5H)-Furanone	C_4_H_4_O_2_	497-23-4	Buttery
7.	2,3-dimethylpyrazine	C_6_H_8_N_2_	5910-89-4	Nutty nut skin cocoa peanut butter coffee walnut caramellic roasted
8.	2-methyl butyraldehyde	C_5_H_10_O	96-17-3	Musty cocoa phenolic coffee nutty malty fermented fatty alcoholic
9.	2-propionyl pyrrole	C_7_H_9_NO	1073-26-3	Roast popcorn
10.	3-hepten-2-one	C_7_H_12_O	1119-44-4	Green grassy caraway
11.	(Z)-3-nonenal	C_9_H_16_O	31823-43-5	Cucumber
12.	3-pentanone	C_5_H_10_O	96-22-0	Ethereal acetone
13.	4-ethyl phenol	C_8_H_10_O	123-07-9	Phenolic castoreum smoke guaiacol
14.	Acetoin	C_4_H_8_O_2_	513-86-0	Sweet buttery creamy dairy milky fatty
15.	Alpha-Phellandrene	C_10_H_16_	99-83-2	Citrus herbal terpene green woody peppery
16.	Benzophenone	C_13_H_10_O	119-61-9	Balsam rose metallic powdery geranium
17.	Benzyl alcohol	C_7_H_8_O	100-51-6	Floral rose phenolic balsamic
18.	Beta-Pinene	C_10_H_16_	127-91-3	Dry woody resinous pine hay green
19.	Cyclohexanol	C_6_H_12_O	108-93-0	Camphor menthol phenol
20.	Delta-nonalactone	C_9_H_16_O_2_	3301-94-8	Coconut creamy sweet milky coumarin
21.	Delta-undecalactone	C_11_H_20_O_2_	710-04-3	Creamy fatty coconut fruity peach waxy
22.	Dimethyl sulfoxide	C_2_H_6_OS	67-68-5	Fatty oily cheesy garlic mushroom
23.	Dipentène / terpène/limonene	C_10_H_16_	68956-56-9	Pine citrus herbal green
24.	Ethyl maltol	C_7_H_8_O_3_	4940-11-8	Sweet caramel jam strawberry cotton candy
25.	Ethyl propanoate	C_5_H_10_O_2_	105-37-3	Sweet fruity rum juicy fruit grape pineapple
26.	Ethylbenzene	C_8_H_10_	100-41-4	Floral plant sweet
27.	Gamma-decalactone	C_10_H_18_O_2_	706-14-9	Fresh oily waxy peach coconut buttery sweet
28.	Hexanoic acid	C_6_H_12_O_2_	142-62-1	Sour fatty sweat cheese
29.	Methyl 2-methylbutanoate	C_6_H_12_O_2_	868-57-5	Ethereal estery fruity tutti frutti green apple lily of the valley powdery fatty
30.	Methyl decanoate	C_11_H_22_O_2_	110-42-9	Oily wine fruity floral
31.	Methyl eugenol	C_11_H_14_O_2_	93-15-2	Sweet fresh warm spicy clove carnation cinnamon
32.	Methyl heptanoate	C_8_H_16_O_2_	106-73-0	Sweet fruit green orris waxy floral berry
33.	Octadecanal	C_18_H_36_O	638-66-4	Oily
34.	Propan-2-one	C_3_H_6_O	67-64-1	Solvent ethereal apple pear
35.	Triethyl phosphate	C_6_H_15_O_4_P	78-40-0	Mild cider
36.	Carbon disulfide	CS_2_	75-15-0	Aromatic flavor, burnt scorched fruit, sulfur-containing sweet
37.	1R-(+)-alpha-pinene	C_10_H_16_	7785-70-8	Harsh terpene aromatic minty
38.	L-(−)-alpha-pinene	C_10_H_16_	7785-26-4	Sharp, warm, resinous, fresh pine
39.	Octadienone	C_8_H_12_O	65767-22-8	Geranium metallic
40.	2-Propionylpyrrole	C_7_H_9_NO	1073-26-3	Roast popcorn
41.	Phenol, 2-methyl-	C_7_H_8_O	95-48-7	Musty phenolic plastic medicinal herbal leathery
42.	Octadecane	C_18_H_38_	593-45-3	Fruit fuel flavor fusel alcohol sweet
43.	Propanal	C_3_H_6_O	123-38-6	Earthy alcohol wine whiskey cocoa nutty
44.	Ethanol	C_2_H_6_O	64-17-5	Strong alcoholic ethereal medical
45.	3-Methylfuran	C_5_H_6_O	930-27-8	-
46.	delta-Tetradecalactone	C_14_H_26_O_2_	2721-22-4	Waxy creamy buttery oily fatty cheesy milky dairy
47.	4-vinylguaiacol	C9H10O2	7786-61-0	Sweet spicy clove carnation phenolic peppery smoky woody powdery
48.	Hexylcyclopentane	C_11_H_22_	4457-00-5	-
49.	1-Octen-3-ol	C_8_H_16_O	3391-86-4	Mushroom, earthy, green, oily, fungal raw chicken

“-” means no obvious odor.

#### 3.5.2 Color difference analysis

**Figure 7:**
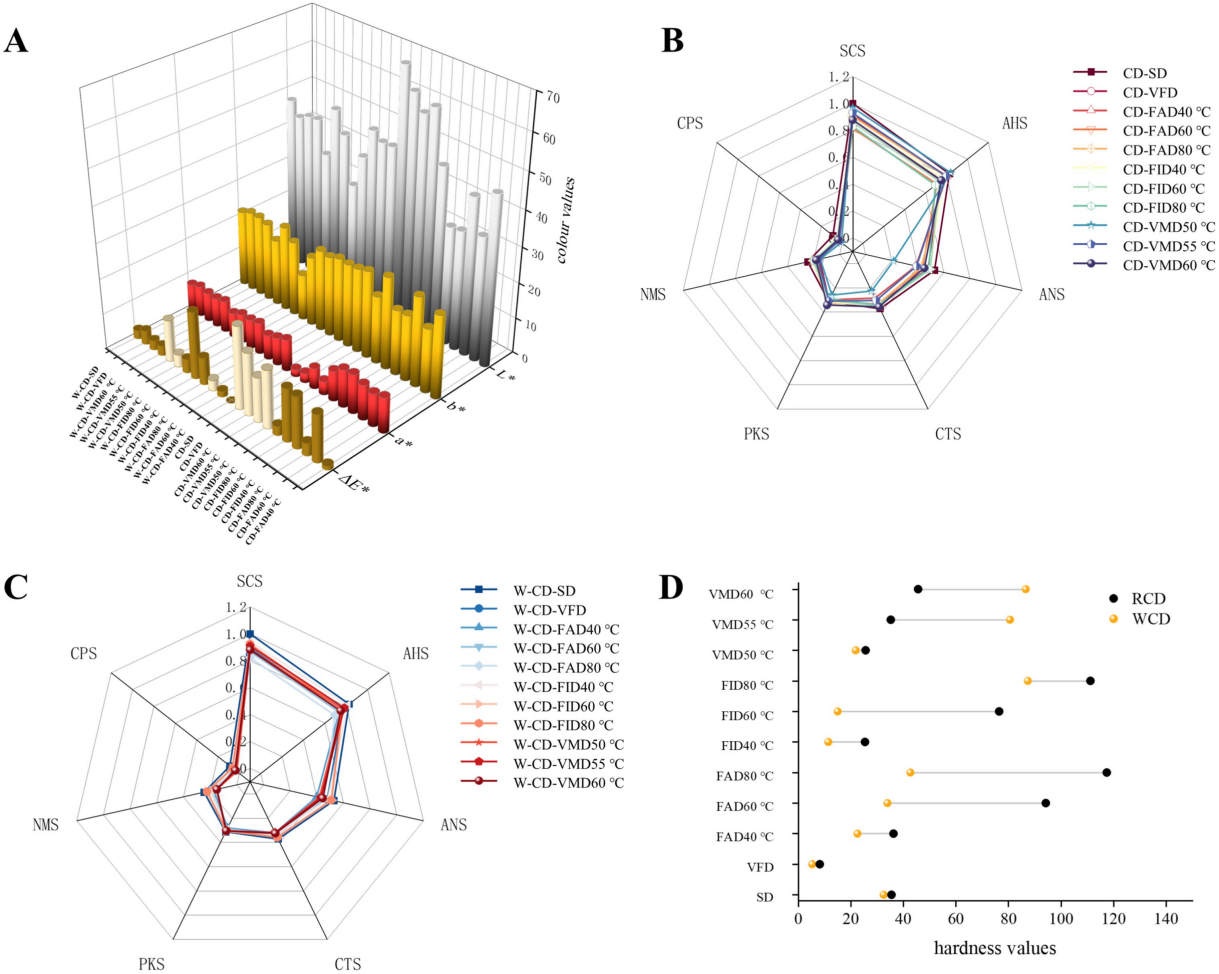
The color difference **(A)**, E-tongue taste analysis (**B**: CD; **C**: W-CD), and hardness analysis **(D)** of CD and W-CD.

Color characteristics were performed by using CM-5-differential refractometer and the results of L^*^-value, a^*^-value, b^*^-value and ΔE^*^ were shown in Figure 7A. As seen in a^*^-value of CD and W-CD in vacuum-dried (VFD and VMD) were 2.03 ~ 5.83 to compare other samples 6.97 ~ 11.76 in SD, FAD, FID method that was suggested this dry method effects on the red color intensity of samples. The vacuum-dried can effectively retain the original color of CD and W-CD, as indicated by the L^*^-value and b^*^-value. Combined with ΔE^*^ analysis, the different drying conditions significantly affect the color, with the sequence being VFD > VMD > SD > FAD > FID of CD, and SD > W-VFD > W-VMD > W-FAD > W-FID of W-CD. Notably, these results demonstrate that the color was significantly changed by the rice wine-steaming process; the L^*^-value, a-value, and b^*^-value were combined with taste and hardness characteristics of all samples to investigate the important distinguishing factor between them.

#### 3.5.3 E-tongue taste analysis

To explore the taste features of CD and W-CD under different drying methods, the AHS, ANS, SCS, CTS, and NMS were performed, as shown in Figures 7B,C. The taste profile of CD was SCS > AHS > ANS > CTS > NMS (Figure 7B), and W-CD was ANS > SCS > AHS > CTS > NMS (Figure 7C). The bitterness and sourness of CD were reduced while sweetness was increased via rice wine-steamed processing. Moreover, different dry methods can also influence the taste of CD and W-CD. The taste characteristics of the samples were used in chemometrics research to comprehensively analyze the quality differences in the following research.

#### 3.5.4 Hardness of CD and rice wine-steamed CD

**Figure 8:**
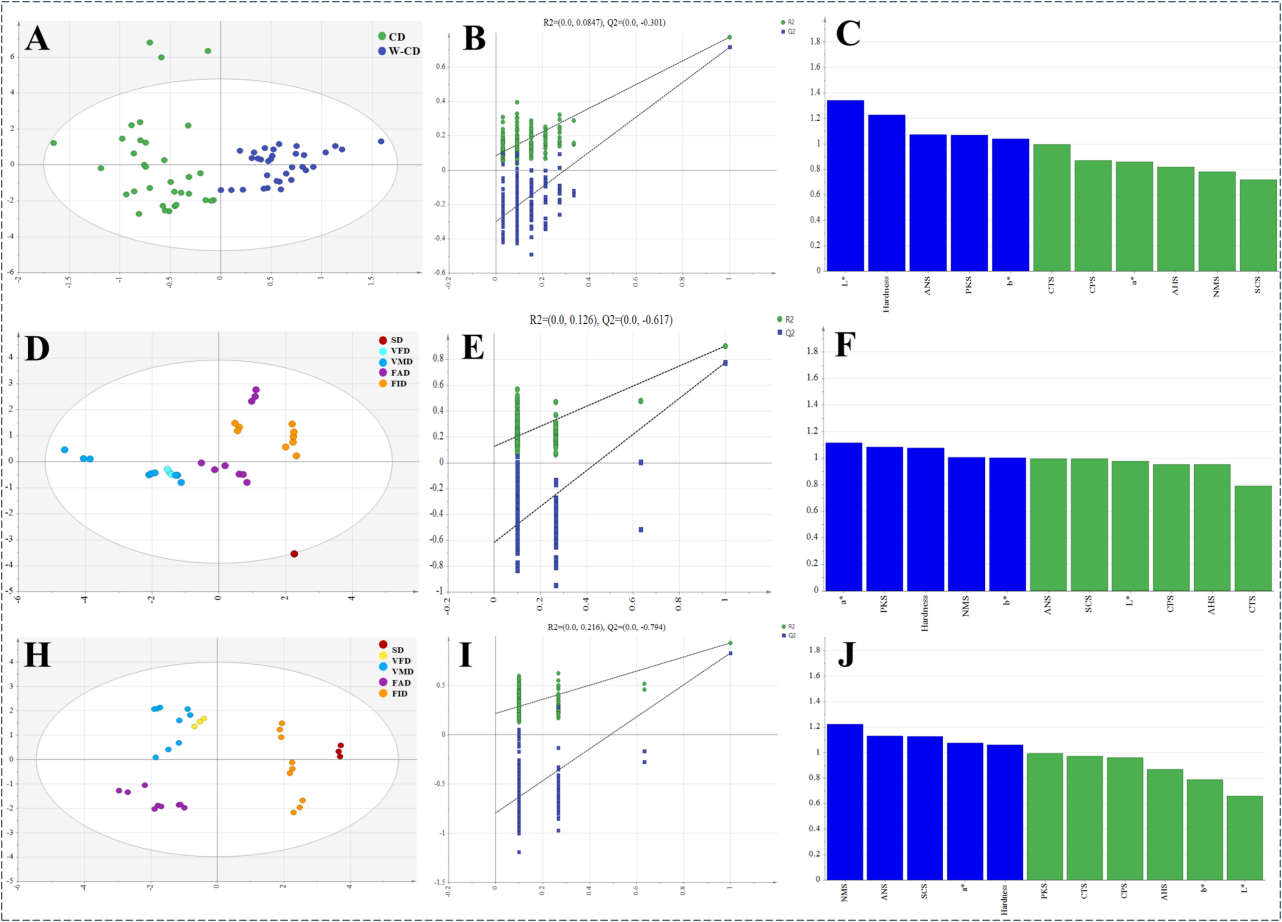
Color characteristics, tastiness, and hardness of different processing methods of CD and W-CD. The highlight is changed to: The PLS-DA analysis **(A)**, permutation test **(B)**, and VIP analysis (C) of CD and W-CD; PLS-DA analysis **(D)**, permutation test **(E)**, and VIP analysis **(F)** of CD in different dry methods; PLS-DA analysis **(H)**, permutation test **(I)**, and VIP analysis **(J)** of W-CD in different dry methods.

Moreover, does the texture of the sample change due to different drying methods? Therefore, the hardness of CD and W-CD was investigated. As depicted in Figure 7D, the CD and W-CD of VFD were crisp and had the lowest hardness, whereas the FAD products had the highest hardness. It was known that the appearance changed obviously, including the color, odor, taste, and hardness of CD and W-CD. Furthermore, above those, chemometric analyses were used to differentiate variables, and PLS-DA analysis classified the different products (Figures 8A,D,H). Permutation tests showed that the model was not overfitting (Figures 8B,E,I). VIP > 1 of L^*^, b^*^, hardness, ANS, and PKS were distinguished CD and W-C (Figure 8C). Similarly, the a^*^, NMS, and hardness were important factors for the identification of different drying products of CD and W-CD (Figures 8B,C). Overall, these results demonstrated that it was reliable to distinguish products by examining their appearance characteristics.

### 3.6 AHP analysis of contents of CD and rice wine-steamed CD

The AHP method was used to further determine the best drying process for CD and W-CD. In processing characteristics, the nine key quality evaluation indicators were divided into five levels. The priority order of each indicator was determined: drying time (*Y_1_*) > echinacoside (*Y_2_*) = verbascoside (*Y_3_*) > extracts (*Y_4_*) > polysaccharides (*Y_5_*) > cistanoside A (*Y_6_*) = tubuloside A (*Y_7_*) = Isoacteoside (*Y_8_*) = 2′-acetylverbascoside (*Y_9_*). The judgment matrix was constructed for consistency testing and shown in Supplementary Tables S9, S10, where the consistency ratio (CR, %) was 0.0083 < 0.1, indicating that the weight distribution was reasonable and reliable. Importantly, the comprehensive scores of the samples were evaluated for making the data unified and normalized, which follows as: (0.2736*Y_1_* + 0.1801*Y_2_* + 0.1801*Y_3_* + 0.1167*Y_4_* + 0.0744*Y_5_* + 0.0438*Y_6_* + 0.0438*Y_7_* + 0.0438*Y_8_* + 0.0438*Y_9_*) × 100. The results are shown in Supplementary Table S10; the comprehensive AHP score indicated that the CD at FAD60-80 °C was the best. The FID 40 °C was a great method for producing W-CD.The scores of these drying methods were above 70.

### 3.7 Correlation analysis

**Figure 9:**
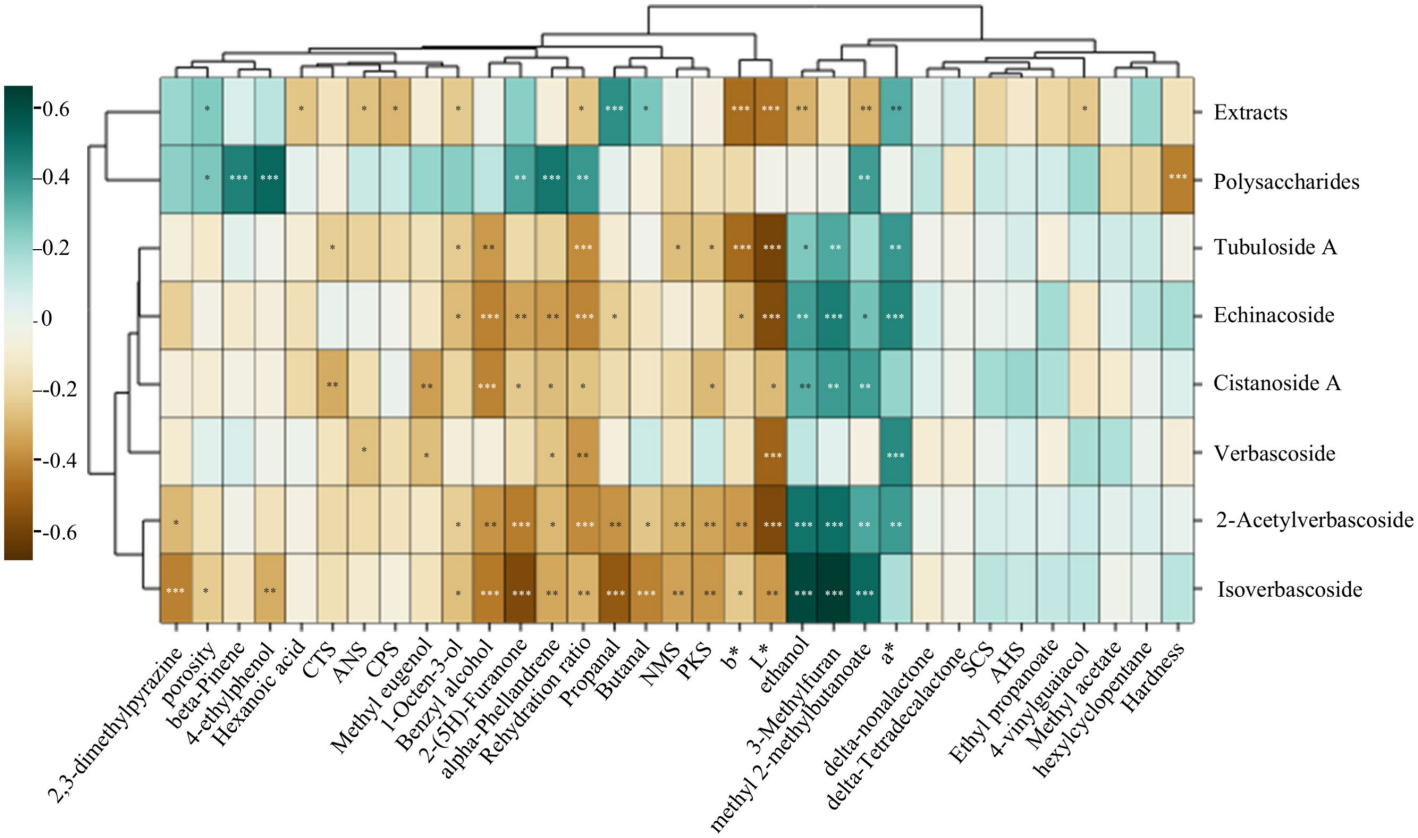
Correlation clustering heat map (*p < 0.05, **p < 0.01, ***p < 0.001).

In this study, correlation analysis between the indicators was employed to explore association rules between appearance traits (odor, hardness, color, taste, porosity, and RR) and internal components (content of phenylethanoid glycoside, total polysaccharide, and extracts) of the CD and W-CD. As shown in Figure 9, both the a*-value and ethanol, methyl 2-methylbutanoate were positively correlated with phenylethanoid glycoside content. The porosity, RR, methyl 2-methylbutanoate, 2-(5H)-Furanone, alpha-Phellandrene, beta-Pinene, and 4-ethyl phenol were also positively correlated with phenylethanoid glycoside content, while hardness was negatively correlated. Furthermore, L*-value, b*-value, ANS, CPS, hexanoic acid, 1-Octen-3-ol, methyl 2-methylbutanoate, and 4-vinylguaiacol were negatively correlated with extract content. Both the higher a* value and the lower L* and b* values indicate a higher component in the sample. The higher the contents of ethanol, 3-Methylfuran, and methyl 2-methylbutanoate, the higher the content of phenylethanoid glycosides and total polysaccharide. Hence, it has been speculated that intelligent sensory technology could enable fast detection of quality discrimination between CD and W-CD.

## 4 Discussion

CD was a very beneficial plant for humans, whether used as food or medicine. The processing method was one of the most important factors influencing the quality of CD and W-CD. In this study, the effects of different drying methods on fresh CD and W-CD were investigated to compare their quality using a comprehensive analysis of “color—odor—taste—component content.” Weibull functions were used to fit the dynamic drying process to evaluate and simulate application scenarios for different drying methods in actual production. In this process, the DR was an important index for measuring the drying efficiency of materials, and was the amount of water removed per unit time (29). During the drying process, D_eff_ describes the rate at which water diffuses from within the material to its surface. D_eff_ was proportional to drying temperature, drying time, and DR. The greater the D_eff_ value, the faster the diffusion rate (25). Cause RR was regularly used as an index for evaluating the quality of dried products that reflect the degree of denaturation based on the material’s ability to reabsorb water during the drying process (30), and the higher the RR, the better the quality under the same drying conditions (31). Above these, VMD reduces the time by about 98% compared with other methods, which significantly improves the drying rate and saves drying time of CD and W-CD.

During TCM processing, different drying methods significantly affect the content of chemical components (32, 33). Rice wine-steamed proccessing CD can improve the content of phenylethanoid glycosides. The CD-VFD slices had the highest polysaccharide content, and CD-VMD had the highest total extract. Herein, E-tongue and color difference meter analysis played an important role in sensory and quality evaluation of food and herb medicine (34–37). The ΔE value of CD-VFD was the smallest, the closest color to the fresh samples. Importantly, the sweetness (ANS) was increased after rice wine-steaming of CD. Furthermore, floral, fruity, grassy, and minty odors were distinguished using an E-nose to analyze in CD and W-CD. Combined with chemometrics analysis, electronic sensory technology was a strategy for discriminating between CD and W-CD. Moreover, AHP can consider factors of different dimensions in the decision-making process to form a hierarchical structure, to select the standard to choose the best solution (38). This decision-making process involves multiple dimensions, including drying time and some internal components. The best drying condition for CD in FAD60-80 °C, and W-CD in FID 40 °C. In summary, the drying methods used to process fresh CD and W-CD were preferred in this study. Improving the quality of CD and avoiding the waste of resources, suggesting that the direct processing of fresh CD was necessary. Rice wine-steamed proccessing CD can enhance the product’s taste. In addition to the related products of CDs and W-CDs need further exploitation and research. Our research provides a basis for promoting the standardization of drying methods in the large-scale production of the TCM industry and for constructing a standardized industrial chain of fresh processing and further drying of TCM decoction pieces.

## 5 Conclusion

Our findings revealed that phenylethyl glycoside and the sweetness (ANS) were markedly increased via rice wine-steaming of CD. The drying function model of CD and W-CD was established for different drying methods. The drying method significantly affects the appearance and internal structure of CD and W-CD. The best drying condition for CD was FAD60-80 °C, and for W-CD was FID40 °C. Moreover, intelligent sensory (color difference meter, e-nose, and e-tongue) systems were expected to develop into a rapid discrimination for CD and W-CD. Taken together, this paper can support a basis for the research and development of CD in food, medicine, or health food.

## Data Availability

The original contributions presented in the study are included in the article/Supplementary material, further inquiries can be directed to the corresponding author.

## Glossary

AHP: Analytic Hierarchy Process
AHS: sourness
ANS: sweetness
CCA: canonical correlation analysis
CD: *Cistanche deserticolac*
CTS: saltness
D_eff_: Effective moisture diffusion coefficient
DR: Drying rate
E_a_: drying activation energy
FAD: forced air drying
FID: far-infrared drying
MR: moisture ratio
M_t_: dry basis moisture content
NMS: freshness
PLS-DA: partial least squares discriminant analysis
PCA: principal component analysis
RMSE: root mean square error
RR: Rehydration ratio
SCS: bitterness
SD: sun drying
TCM: traditional Chinese medicine
VFD: vacuum freeze drying
VIP: Variable Importance Projection
VMD: vacuum microwave drying
VOCs: volatile components
W-CD: rice wine-steamed CD
