# Supplementary material for: Quality evaluation of Cistanche deserticola and rice wine-steamed products: drying kinetics, intelligent sensory, and chemometrics analysis
Source: Front Nutr. 2026 Jan 27;12:1732810. doi: 10.3389/fnut.2025.1732810 (PMC12888225; doi:10.3389/fnut.2025.1732810)
Supplement: Supplementary file 1 [file Table_1.docx]

**Quality evaluation of *Cistanche deserticola* and yellow rice wine-steamed products: drying kinetics, intelligent sensory and** **chemometrics analysis**

Zhangli Jiang ^a,b,c,^ ^†^, Shiyuan Tang ^a,b,c,^ ^†^, Xu Wu ^a,b,c,^ ^†^, Hui Zhang ^a,b,c^, Xinyi Zhang ^a,b,c^, Zihan Ma ^a,b,c^, Xiaohui Bian ^a,b,c^, Hui Wang ^a,b,c^, Xin Chai ^d,e^, Yuefei Wang ^d,e^, Zhiying Dou ^a,b,c^ *

1. School of Chinese Materia Medica, Tianjin University of Traditional Chinese Medicine, Tianjin 301617, China.
2. Traditional Chinese Medicine Processing Techniques Heritage Base (Tian jin), National Administration of Traditional Chinese Medicine, Tianjin 301617, China.
3. National Inheritance Studio of Expert Chinese Materia Medica, Tianjin 301617, China.
4. State Key Laboratory of Chinese Medicine Modernization, Tianjin University of Traditional Chinese Medicine, Tianjin, 301617, China
5. Haihe Laboratory of Modern Chinese Medicine, Tianjin, 301617, China

* Corresponding author: Prof. Zhi-Ying Dou, School of Chinese Materia Medica, Tianjin University of Traditional Chinese Medicine, No. 10 Poyang Lake Road, West Tuan Bo New Town, Jing Hai District, 301617, P.R. China. Tel/Fax: (022)23052241. E-mail address: zhiyingdou@tjutcm.edu.cn (Z. Y. Dou).

^†^ These authors have contributed equally to this work.

Supplementary table1 Linear regression for the calibration curve of Phenylethanoid glycosides (n=6)

| Compounds | Calibration curve | R^2^ | Linear range  (μg /mL) |
| --- | --- | --- | --- |
| Echinacoside | *Y* = 12561*X* - 68.673 | 0.9999 | 10.80～1008.00 |
| Cistanoside A | *Y* = 12072*X* - 3.2596 | 0.9998 | 0.80～99.00 |
| Tubuloside A | *Y* = 12564*X* - 7.1421 | 0.9998 | 0.50～114.00 |
| Verbascoside | *Y* = 15519*X* - 19.026 | 0.9998 | 3.90～196.00 |
| Isoverbascoside | *Y* = 14362*X* - 12.506 | 0.9998 | 1.60～203.00 |
| 2’-Acetylverbascoside | *Y* = 13319*X* - 3.2239 | 0.9998 | 0.50～61.00 |

Supplementary table2 Precision of each component in Phenylethanoid glycosides (n=6)

| Compounds | Time (min) | Average peak area | RSD (%) |
| --- | --- | --- | --- |
| Echinacoside | 14.4 | 5095.50 | 0.93 |
| Cistanoside A | 17.1 | 645.12 | 0.60 |
| Tubuloside A | 19.1 | 69.32 | 3.64 |
| Verbascoside | 20.06 | 534.92 | 0.96 |
| Isoverbascoside | 23.06 | 602.15 | 0.66 |
| 2’-Acetylverbascoside | 28.9 | 102.05 | 0.58 |

Supplementary table3 Stability of each component in Phenylethanoid glycosides (n=6)

| Compounds | Average peak area | RSD (%) |
| --- | --- | --- |
| Echinacoside | 4990.89 | 1.63 |
| Cistanoside A | 615.89 | 0.87 |
| Tubuloside A | 72.93 | 2.18 |
| Verbascoside | 529.94 | 1.13 |
| Isoverbascoside | 598.63 | 0.89 |
| 2’-Acetylverbascoside | 92.97 | 2.50 |

Supplementary table4 Repeatability of each component in Phenylethanoid glycosides (n=6)

| Compounds | Average content (mg/g) | RSD (%) |
| --- | --- | --- |
| Echinacoside | 20.54 | 0.72 |
| Cistanoside A | 2.42 | 0.40 |
| Tubuloside A | 0.30 | 1.95 |
| Verbascoside | 1.83 | 1.26 |
| Isoverbascoside | 2.04 | 1.86 |
| 2’-Acetylverbascoside | 0.33 | 0.16 |

Supplementary table 5 Linear regression for the calibration curve of Total polysaccharides (n=6)

| Compounds | Calibration curve | R^2^ | Linear range (μg /mL) |
| --- | --- | --- | --- |
| Total polysaccharides | *y* = 15.486*x* + 0.1332 | 0.9994 | 0.74～95.00 |

Supplementary table 6 Precision of each component in Total polysaccharides (n=6)

| Compounds | Absorbance | Average Absorbance | RSD (%) |
| --- | --- | --- | --- |
| Total polysaccharides | 0.351 | 0.351 | 0.12 |

Supplementary table 7 Stability of each component in Total polysaccharides (n=6)

| Compounds | Average Absorbance | RSD (%) |
| --- | --- | --- |
| Total polysaccharides | 0.352 | 0.34 |

Supplementary table 8 Repeatability of each component in Total polysaccharides (n=6)

| Compounds | Average content (mg/g) | RSD (%) |
| --- | --- | --- |
| Total polysaccharides | 56.16 | 1.58 |

Table 9 Weight index pairwise comparison judgment priority matrix

| Indicators | Drying time | Echinacoside | verbascoside | Extracts | Polysaccharides | Cistanoside A | Tubuloside A | Isoacteoside | 2’-acetylverbascoside | ωi |
| --- | --- | --- | --- | --- | --- | --- | --- | --- | --- | --- |
| Drying time | 1 | 2 | 2 | 3 | 4 | 5 | 5 | 5 | 5 | 0.2736 |
| Echinacoside | 1/2 | 1 | 1 | 2 | 3 | 4 | 4 | 4 | 4 | 0.1801 |
| verbascoside | 1/2 | 1 | 1 | 2 | 3 | 4 | 4 | 4 | 4 | 0.1801 |
| Extracts | 1/3 | 1/2 | 1/2 | 1 | 2 | 3 | 3 | 3 | 3 | 0.1167 |
| Polysaccharides | 1/4 | 1/3 | 1/3 | 1/2 | 1 | 2 | 2 | 2 | 2 | 0.0744 |
| Cistanoside A | 1/5 | 1/4 | 1/4 | 1/3 | 1/2 | 1 | 1 | 1 | 1 | 0.0438 |
| Tubuloside A | 1/5 | 1/4 | 1/4 | 1/3 | 1/2 | 1 | 1 | 1 | 1 | 0.0438 |
| Isoacteoside | 1/5 | 1/4 | 1/4 | 1/3 | 1/2 | 1 | 1 | 1 | 1 | 0.0438 |
| 2’-acetylverbascoside | 1/5 | 1/4 | 1/4 | 1/3 | 1/2 | 1 | 1 | 1 | 1 | 0.0438 |

Table 10 Result of comprehensive scores

| Sample name | Drying time | Echinacoside | Verbascoside | Extracts | Polysaccharides | Cistanoside A | Tubuloside A | Isoacteoside | 2’-acetylverbascoside | comprehensive scores | Rank Result |
| --- | --- | --- | --- | --- | --- | --- | --- | --- | --- | --- | --- |
| CD-FAD40 °C | 0.52 | 0.34 | 0.09 | 0.13 | 0.42 | 1.00 | 0.16 | 0.22 | 0.19 | 33.42 | 7 |
| CD-FAD60 °C | 0.72 | 1.00 | 0.74 | 0.70 | 0.22 | 0.60 | 0.64 | 0.6 | 1.00 | 73.48 | 2 |
| CD-FAD80 °C | 0.83 | 0.64 | 1.00 | 0.94 | 0.15 | 0.30 | 1.00 | 1.00 | 0.85 | 78.17 | 1 |
| CD-FID40 °C | 0.00 | 0.59 | 0.21 | 0.35 | 0.48 | 0.54 | 0.66 | 0.00 | 0.49 | 29.43 | 10 |
| CD-FID60 °C | 0.50 | 0.26 | 0.10 | 1.00 | 0.42 | 0.01 | 0.13 | 0.13 | 0.24 | 37.23 | 4 |
| CD-FID80 °C | 0.73 | 0.26 | 0.16 | 0.04 | 0.20 | 0.34 | 0.17 | 0.35 | 0.31 | 34.43 | 6 |
| CD-VMD50 °C | 0.99 | 0.00 | 0.00 | 0.34 | 0.00 | 0.00 | 0.00 | 0.00 | 0.00 | 31.17 | 9 |
| CD-VMD55 °C | 1.00 | 0.55 | 0.05 | 0.24 | 0.02 | 0.32 | 0.02 | 0.00 | 0.00 | 42.48 | 3 |
| CD-VMD60 °C | 1.00 | 0.11 | 0.09 | 0.13 | 0.03 | 0.11 | 0.03 | 0.00 | 0.00 | 33.32 | 8 |
| CD-VFD | 0.70 | 0.18 | 0.06 | 0.10 | 1.00 | 0.52 | 0.07 | 0.18 | 0.14 | 35.99 | 5 |
| CD-SD | 0.39 | 0.30 | 0.27 | 0.00 | 0.30 | 0.23 | 0.19 | 0.14 | 0.35 | 27.30 | 11 |
| W-CD-FAD40 °C | 0.37 | 0.00 | 0.22 | 0.88 | 0.27 | 0.09 | 0.41 | 0.01 | 0.31 | 30.06 | 10 |
| W-CD-FAD60 °C | 0.66 | 0.76 | 0.44 | 0.27 | 0.52 | 1.00 | 0.24 | 0.45 | 0.41 | 55.88 | 3 |
| W-CD-FAD80 °C | 0.76 | 0.32 | 0.04 | 0.56 | 0.25 | 0.00 | 0.41 | 0.02 | 0.00 | 37.50 | 6 |
| W-CD-FID40 °C | 0.00 | 1.00 | 1.00 | 1.00 | 1.00 | 0.87 | 1.00 | 1.00 | 1.00 | 72.09 | 1 |
| W-CD-FID60 °C | 0.43 | 0.94 | 0.00 | 0.14 | 0.22 | 0.60 | 0.27 | 0.00 | 0.07 | 36.01 | 8 |
| W-CD-FID80 °C | 0.63 | 0.56 | 0.16 | 0.17 | 0.00 | 0.36 | 0.00 | 0.52 | 0.27 | 37.08 | 7 |
| W-CD-VMD50 °C | 1.00 | 0.36 | 0.58 | 0.34 | 0.12 | 0.45 | 0.39 | 0.75 | 0.54 | 58.47 | 4 |
| W-CD-VMD55 °C | 1.00 | 0.56 | 0.33 | 0.23 | 0.14 | 0.37 | 0.20 | 0.48 | 0.38 | 53.40 | 5 |
| W-CD-VMD60 °C | 1.00 | 0.64 | 0.7 | 0.12 | 0.15 | 0.25 | 0.57 | 0.80 | 0.61 | 63.77 | 2 |
| W-VFD | 0.46 | 0.22 | 0.36 | 0.09 | 0.30 | 0.19 | 0.30 | 0.50 | 0.32 | 31.97 | 9 |
| W-SD | 0.25 | 0.67 | 0.01 | 0.00 | 0.54 | 0.48 | 0.02 | 0.09 | 0.01 | 25.92 | 11 |
